# Can We Barter Local Taxes for Maintaining Our Green? A Psychological Perspective

**DOI:** 10.3389/fpsyg.2022.816217

**Published:** 2022-02-22

**Authors:** Annalisa Theodorou, Angelo Panno, Mariagrazia Agrimi, Emanuela Masini, Giuseppe Carrus

**Affiliations:** ^1^Department of Education, Roma Tre University, Rome, Italy; ^2^Department of Human Science, European University of Rome, Rome, Italy; ^3^Department for Innovation in Biological, Agro-Food and Forest Systems, University of Tuscia, Viterbo, Italy

**Keywords:** urban ecosystem service, urban ecosystem disservice, local law, urban green, green maintenance, civic participation, residents’ involvement, neighborhood participation

## Abstract

Previous research highlighted that the desire for neighborhood improvement is an antecedent of the citizens’ involvement in green urban areas maintenance. Nevertheless, the topic of civic participation in the maintenance of green areas is not yet well developed in the literature and a link with local legislation is missing. We investigate the intention of participation in such maintenance through a web-based experiment. We hypothesize that stimuli of poor (vs. good) maintenance will be associated with a higher intention of contributing to the upkeep of green areas following the administrative barter law. The administrative barter is a law approved in Italy, which gives citizens the possibility of a reduction of local taxes in exchange for their involvement in the improvement of the territory. One hundred ninety-six participants (*M*_age_ = 33.81) were assigned randomly to good maintenance condition (*n* = 100) or poor maintenance condition (*n* = 96). The level of maintenance was manipulated through photographs of a neighborhood depicting good or poor maintenance of the urban green ornamentation. Results pointed out that people showed a greater willingness to engage in the improvement of green urban areas in the poor condition as compared to the good condition, according to the administrative barter law. This study suggests that local legislation may provide an incentive fostering citizens’ involvement in green urban areas maintenance.

## Introduction

The cities’ landscapes are changing as the awareness of the importance of green in urban areas for people’s lives is increasing. The trees of avenues, parks, and gardens, originally created for ornamental purposes, now play an important role in urban life for their ability to enhance individuals’ physical health as well as socio-psychological well-being (Tzoulas et al., 2007; van Dillen et al., 2012). Urban greenspace has been associated with greater physical activity and longevity (van Dillen et al., 2012; Rojas-Rueda et al., 2019). Moreover, exposure to natural views has been related to greater psychological well-being with lower stress symptoms and depression, and greater attention (Li and Sullivan, 2016; Braçe et al., 2020). The supportive effect of green views for mental health was particularly evident during Covid-19 lockdowns (Soga et al., 2021). Nevertheless, the benefits are not limited to the individual alone; indeed, the presence of natural elements in the urban ecosystem was found to enhance a sense of community, social relationships among residents, and even to lower criminality (Kuo and Sullivan, 2001; Donovan and Prestemon, 2012; van Dillen et al., 2012). Finally, benefits for residents pass also through the role of urban forests and trees as Nature-Based Solutions, namely adaptation strategies to many urban environmental problems, such as local cooling, air pollution reduction, and urban stormwater runoff management (Mullaney et al., 2015).

Generally speaking, the importance of green urban spaces is recognized and valued by citizens (Grima et al., 2020). However, studies suggested that one factor that can limit the benefits of such areas is the state of maintenance (von Döhren and Haase, 2015). Poor upkeep can hinder the visit to urban parks and also diminish the willingness to engage in physical activity in green areas (Van Hecke et al., 2018). On the contrary, good maintenance of urban green beautification seems to impact the preference for an area (Kuo et al., 1998). According to the literature, physical aspects of the neighborhood such as esthetics, walkability, and, importantly, state of maintenance are also linked to perceptions of safety and even provide insight into the relationships between area residents (Kuo et al., 1998; Cerin et al., 2006; O’Brien and Wilson, 2011). Individuals use environmental cues adaptively, remaining vigilant in a neighborhood deemed potentially dangerous. They are therefore attentive and read the information that comes from the physical conditions of a neighborhood as they are used to program their behavior (O’Brien and Wilson, 2011; Donovan and Prestemon, 2012). Following this line, well-maintained areas convey a sense of community care, which in turn is supposed to prevent the engagement in criminal behavior (Kuo and Sullivan, 2001).

Overall, we can define maintenance as an urban ecosystem service and poor upkeep as an urban ecosystem disservice (Millennium Ecosystem Assessment, 2005; von Döhren and Haase, 2015). Urban ecosystem services refer to the benefits of natural ecosystems, while urban ecosystem disservices origin from the qualities of the natural environment that are perceived as harmful, unpleasant, or unwanted (Millennium Ecosystem Assessment, 2005; Shapiro and Báldi, 2014; Villa et al., 2014; Lyytimäki, 2015; von Döhren and Haase, 2015). According to some authors, this classification encourages discussion about the social and economic consequences of disservices (Shapiro and Báldi, 2014). Roots coming out of the streets can lead to trips, long branches can cover important traffic signs, undesired wild vegetation can contribute to a loss of esthetics of the landscape as well as instill a sense of insecurity. These are all examples of poor upkeep that can lead to discomforts. Furthermore, when maintenance is procrastinated or even absent, it can lead to damage to the adjacent environment (e.g., tarmac, water pipes), with subsequent need for additional public funding (von Döhren and Haase, 2015).

In sum, green urban spaces are central to the life of urban residents, but they are not always in a good condition to ensure the benefits they can bring to people (von Döhren and Haase, 2015). Therefore, it becomes important to investigate how to support it. Hence, what can be done to sustain maintenance? Research suggests that a key role is played by the involvement of residents (Ohmer et al., 2009). The upkeep of gardens, parks, and green decorations is often sustained by volunteers and neighbors’ associations (Wolf et al., 2013). However, in a study, it was found that although citizens report avoiding visiting parks when they feel there had been improper management, only 20% of them would like to be involved in the maintenance (Lee and Kim, 2015). In some countries, local administrations have issued laws that can stimulate civic participation. For instance, the administrative barter is an Italian law that guarantees reduction or exemptions from local taxes to people who want to participate in the maintenance of the urban green areas (Cepiku, 2017). Nevertheless, participation in such initiatives is increasingly diminishing over the years (Istat, 2021, 2018).

It is important to note that engagement and participation result in economic advantage, contributing also to the improvement of governance (Butt et al., 2021). Therefore, the focus should be on the factors that can promote engagement. Previous findings point to the desire for neighborhood improvement as an antecedent of citizens’ involvement in green urban areas maintenance (Moskell et al., 2010). In particular, it seems that the perception of a compelling necessity and an urgent intervention is what triggers the willingness to be active participants in the restoration of green spaces and landscape beautification (Wolf et al., 2013). Nevertheless, although there is flourishing literature on the psychological underpinnings of civic participation (Albanesi et al., 2007; Marzana et al., 2012; Francescato et al., 2017), the topic of civic participation specifically concerning the maintenance of green areas is not yet well developed in the literature (Krajter Ostoić and Konijnendijk van den Bosch, 2015). Following this line, the purpose of this study is to investigate the intention of participation in such maintenance, focusing on the perceptions of the qualities of the areas rather than on individuals’ characteristics. Accordingly, our study hypothesis is that poor (vs. good) maintenance will be associated with a higher intention of contributing to the upkeep of green areas following the administrative barter law.

## Methods

### Participants and Procedures

To test our hypothesis, we designed a 2 × 2 between-subjects study collecting data from an online survey. All participants were informed of the voluntary nature of their participation in the study and that they could interrupt the questionnaire at any time. After being assured of anonymity, informed consent was collected from all participants. Data were collected through a Google Form, namely a free Google app for creating surveys, shared through social media and personal contacts. There were no particular recruitment targets: the questionnaire was administered to those who understood the Italian language, without limits of age, qualification, or gender. More specifically, there were two versions of the questionnaire, one for each experimental condition (good vs. poor maintenance of the urban green ornamentation), and participants were assigned randomly to the two conditions. The level of maintenance was manipulated through a slideshow of photographs of an Italian neighborhood depicting good or poor maintenance of the urban green ornamentation.

In the condition of poor maintenance, the photographs presented real-life situations where the pavement or the asphalt had been damaged by the roots of trees (see Supplementary Figures 1–4). On the contrary, in the condition of good maintenance, the photographs were modified with a photo editor software to show the good condition of sidewalks and roads (see Supplementary Figures 5–8). Participants were asked to imagine being residents of the neighborhood displayed in the photographs and to answer the questionnaire accordingly. Across the two conditions, the two questionnaires were similar in all aspects except for the presentation of the photographs.

An *a priori* power analysis using the software G*Power (Faul et al., 2007) suggested that a minimum sample size of 169 participants was required to observe, with our study design, an effect size of 0.25 with 90% power at α = 0.05. As recommended by Oppenheimer et al. (2009), to detect participants who were not focusing on the questionnaire, we used one question, a so-called attention check: “*This question is to check the attention of the respondent, if you are paying attention please answer 4*.” Participants who did not answer correctly to the attention check were excluded from the analysis. A total of 220 participants agreed to participate in the research, of which 24 failed to answer the attention check. Thus, our final sample was composed of 196 participants, of which 100 (51%) were in the good maintenance condition and 96 (46%) were in the poor maintenance condition.

Of the total sample, 136 (69.38%) were women. Age ranged from 18 to 81 (M: 33.81; SD = 15.56). Regarding marital status, 129 (65%) were single, 37 (18.9%) were married, 25 (12.8%) were living with the partner, 4 (2%) were divorced, and 1 (0.5%) was widowed. Regarding the education qualification, of the total sample, 11 (5.6%) held a junior high school diploma, 95 (48.5%) held a high school diploma, 30 (15.3%) held a bachelor’s degree, 49 (25%) held a master’s degree, and 11 (5.6%) held a higher degree (e.g., doctoral). Regarding the employment status, 81 (41.3%) were students, 73 (37.2%) were employees, 22 (11.2%) were self-employed, 11 (5.6%) were unemployed, and 9 (4.6%) were pensioners.

### Measures

#### Manipulation Checks

To check if our manipulation was successful, namely if, as expected, the two sets of photos were portraying urban green ornaments in good vs. poor maintenance conditions, we used manipulation check measures. Specifically, we asked participants to evaluate different aspects of the neighborhood shown in the photos, namely pedestrian mobility, road practicability, flooring, and esthetics. The responses were ranked on a 5-point scale.

A one-way ANOVA was performed for each of the four aspects with the condition as the independent variable. As expected, results attested that: 1) pedestrian mobility resulted higher in the good (*M* = 2.10, SD = 0.99) vs. poor (*M* = 1.36, SD = 0.71) maintenance condition and the difference was statistically significant *F*(1, 194) = 35.38, *p* < 0.001, η^2^_*p*_ = 0.15; road practicability was rated higher in the good (*M* = 2.51, SD = 1.07) vs. poor (*M* = 1.84, SD = 0.94) maintenance condition and the difference was statistically significant *F*(1, 194) = 21.13, *p* < 0.001, η^2^_*p*_ = 0.10; flooring was evaluated higher in the good (*M* = 1.73 DS = 0.90) vs. poor (*M* = 1.26, DS = 0.65) maintenance condition and the difference was statistically significant *F*(1, 194) = 17.43, *p* < 0.001, η^2^_*p*_ = 0.08; lastly, esthetics was rated higher in the good (*M* = 1.67 DS = 0.94) vs. poor (*M* = 1.28, DS = 0.69) maintenance condition and the difference was statistically significant *F*(1, 194) = 10.76, *p* < 0.01, η^2^_*p*_ = 0.05. Overall, the manipulation can be considered successful.

#### Willingness to Actively Engage in the Maintenance of the Green Ornamentation of the Neighborhood

After being exposed to the photographs, participants of both conditions were asked to imagine living in the neighborhood shown in the images and answer to a scenario in which they were presented with the possibility to take advantage of the administrative barter law. The administrative barter is a law approved in 2014 in Italy (Law decree n. 164, art. n. 24), which gives citizen or legally recognized associations the possibility of a reduction or exemption of local taxes in exchange for their involvement in the improvement of the territory. We used the following scenario:

In the neighborhood shown in the photos, the law administrative barter is in force (article 24, law 164 of 2014 “Measures to facilitate the participation of local communities in the protection and enhancement of the territory).” According to this law: “Municipalities can define criteria and conditions for the realization of interventions by citizens to enhance the urban or extra-urban territory, such as cleaning, maintenance, embellishment of green areas, squares, streets, or interventions of urban decoration, recovery, and reuse, to act in the general interest, in support of areas and unused real estate. To this end, the local authority may approve the granting of a reduction or exemption of local taxes relating to the activities carried out by the aforementioned subjects.”

After the scenario, participants were asked to answer the following question: *“Based on this law, would you contribute to the adequate maintenance of your neighborhood?”* The possible answers were “Yes” or “No.”

### Statistical Analysis

The data analysis consisted in the analysis of the frequencies of dichotomic responses (yes or no) to the question on the willingness to actively engage in the maintenance of the green ornamentation of the neighborhood. To test the hypothesis that participants in the condition of poor (vs. good) maintenance would report a greater willingness to actively engage in the improvement of green ornaments according to the administrative barter, we proceeded to test whether the proportion of yes and no answers was different by condition (i.e., poor and good maintenance). To do so, we performed the chi-square test for independent samples. This test allows us to compare the “expected” results with those observed in our data. By expected results, in this case, we mean that the yes and no answers that indicate the tendency or not to actively engage in the improvement of green ornaments are distributed in a similar way among both conditions (i.e., poor and good maintenance). In other words, that the yes and no answers are independent of the condition. The higher the chi-square value, the more the observed distribution will differ from that expected. In other words, a high (and significant) chi-squared value will indicate that in the two conditions the proportions of yes and no answers are distributed differently by condition, namely that the two variables are dependent on each other.

## Results

Results attested that the chi-square was significant χ^2^ = (1, 196) = 4.12, *p* < 0.05, suggesting that there was a significant difference between the observed frequency of yes and no responses and the expected one for the two conditions. Table 1 is a contingency table where the yes and no answers are reported by condition: as can be seen, in the condition of poor maintenance we have a greater willingness to actively engage in the improvement of urban green ornaments according to the law of administrative barter as compared to the condition of good maintenance.

**TABLE 1:** Contingency table reporting Yes or No responses to the scenario proposing the administrative barter.

	Willingness to actively engage in the		
	improvement of urban green ornaments		
	No	Yes	Total
Good maintenance	21	79	100
Poor maintenance	10	86	96

Total	31	165	196

## Discussion

The added value of urban greenspace to residents’ lives can be endangered when the areas are not accurately maintained, causing a disservice (von Döhren and Haase, 2015). In this contribution, we wanted to shed light on the motivational mechanisms behind the willingness of the residents to participate in the maintenance of urban greenery in line with the administrative barter law. This regulation establishes that citizens voluntarily engage in the upkeep of their neighborhood in exchange for a deduction in the taxes. To do so, we designed a study in which participants were asked to imagine living in a neighborhood with good vs. poor maintenance of urban green spaces and then indicate whether they would engage in the maintenance following the administrative barter law. Findings revealed that poor upkeep resulted in a higher willingness to participate in the maintenance than good upkeep. These results confirmed our hypothesis.

This study is not free of limitations. First, since data were collected online, results need to be replicated in a more controlled environment such as the laboratory. Second, we captured intentions (a measure sensitive, for instance, to social desirability) and not actual behavior, using a single-item measure. Thus, future studies should replicate findings in the laboratory to directly observe behavior or develop scales to measure intentions. Third, we used a snowball sampling technique that does not always guarantee that individuals in the two groups can be comparable. Fourth, almost half of the participants were students; thus, the generalizability of the findings is limited. Fifth, we used stimuli that focused on the disservices of mostly ornamental trees included in the urban landscape also to implement adaptation strategies to climate change. It will be interesting if future studies could extend the materials presented including images from urban parks. Lastly, we asked participants to imagine being residents of the neighborhood in the pictures. We believe that further studies could examine more closely the intentions of individuals toward their neighborhood since in this relationship other variables could intervene (e.g., place attachment and identity, sense of community).

All in all, our findings are in line with past literature that identified the necessity for maintenance as an important trigger of individuals’ motivation (Moskell et al., 2010; Wolf et al., 2013). Nevertheless, we believe that this research makes a step further. Specifically, it presents innovative aspects that deserve consideration. First, the psychological literature on this specific type of voluntary action is scarce (Moskell et al., 2010). Second, to our knowledge, this is the first psychological study to investigate the willingness to adhere to the administrative barter law. In particular, our study suggests that taxes reduction or exemptions, together with the awareness of poor maintenance, can represent a good incentive that can encourage participation in maintenance. From here, future studies could examine the role of additional variables that could intervene in the relationship observed. For instance, collective efficacy has been proved to play a role in previous studies investigating the willingness to ameliorate one’s neighborhood (Rice et al., 2016). Moreover, motivation can be investigated in line with the literature on voluntarism (Moskell et al., 2010). First, individual differences could explain the participative behavior such as the prosocial personality, psychological empowerment, and political self-efficacy (Penner, 2002; Caprara et al., 2009; Christens et al., 2011). Second, also previous experiences in such types of collective participation can play a role in preventing or stimulating engagement (Moskell et al., 2010).

We believe that this study’s findings offer important practical implications for policymakers. In particular, they point to knowledge and awareness of the state of maintenance as a catalyst of residents’ involvement (Butt et al., 2021). This means that working on enhancing awareness of the poor maintenance and its consequences for the population could promote intentions to change the situation. Apart from the immediate benefits of treating a disservice, it is important to note that involvement in ameliorating one’s community might result also in positive outcomes for residents such as strengthened social relationships with neighbors and an enhanced sense of community and place attachment (Moskell et al., 2010; Stewart et al., 2019).

Lastly, is important to highlight that, apart from the positive implications of the administrative barter law, some authors expressed a few criticisms. The most relevant to our discussion is, in our opinion, the one that considers the exchange proposed by the law as commodification of the right to participate in the maintenance of a collective good as the urban green. In particular, the advocates of such perspective maintain that being more appealing to the disadvantaged citizens, the administrative barter law can be viewed more as an obligation than a free choice, creating dependence and subordination rather than motivating citizens (Giglioni, 2015). Translated in psychological terms, this law would create extrinsic motivation whereas what seems central is how to stimulate intrinsic motivation in residents. We think that working toward a better awareness of both the state of maintenance of green areas and the sociopsychological advantages of civic participation could pave the way to the intrinsic motivation of residents. Another criticism revolves around the issue of political responsibility of the upkeep of public areas and the instrumental use of citizens in the administrative barter law (Giglioni, 2015). Beliefs on political responsibility should be considered, as well as residents’ trust in political institutions. Both variables could play a role in the willingness to engage in the maintenance of green areas following the administrative barter law and, thus, their role should be investigated in future studies.

In conclusion, the implementation of urban forestry and greenery is not always feasible for several reasons (e.g., lack of space); however, when it is included in the city planning, management and upkeep of these areas become vital. It has been proposed that the concept of ecosystem disservice can facilitate the dialog between social sciences (e.g., with the topic of civic participation in the maintenance of green areas) and natural sciences (Lyytimäki et al., 2008). We agree with this conceptualization and focus, in our research, on the contribution of social-environmental psychology to assess what can be done for transforming a malfunction in benefits for individuals. Our study suggests that this can be achieved through combined forces of local administrations and common citizens.

## Data Availability Statement

The raw data supporting the conclusion of this article will be made available by the authors, without undue reservation.

## Ethics Statement

Ethical review and approval was not required for the study on human participants in accordance with the local legislation and institutional requirements. The patients/participants provided their written informed consent to participate in this study.

## Author Contributions

AT and AP contributed to the conception and design of the study. MA and EM contributed to the study materials. AT wrote the first draft of the manuscript, organized the database, and performed the statistical analysis. AP, MA, and EM contributed to the manuscript revision. AP, MA, and GC were the project administrators and acquired the necessary fundings. All authors read and approved the submitted version.

## Conflict of Interest

The authors declare that the research was conducted in the absence of any commercial or financial relationships that could be construed as a potential conflict of interest.

## Publisher’s Note

All claims expressed in this article are solely those of the authors and do not necessarily represent those of their affiliated organizations, or those of the publisher, the editors and the reviewers. Any product that may be evaluated in this article, or claim that may be made by its manufacturer, is not guaranteed or endorsed by the publisher.
