# Supplementary material for: Can We Barter Local Taxes for Maintaining Our Green? A Psychological Perspective
Source: Front Psychol. 2022 Feb 22;13:816217. doi: 10.3389/fpsyg.2022.816217 (PMC8920541; doi:10.3389/fpsyg.2022.816217)
Supplement: Supplementary file 1 [file Presentation_1.pdf]

Supplementary Material to the paper

"Can we barter local taxes for maintaining our green? A psychological perspective"

Annalisa Theodorou, Angelo Panno, Mariagrazia Agrimi, Emanuela Masini, & Giuseppe Carrus

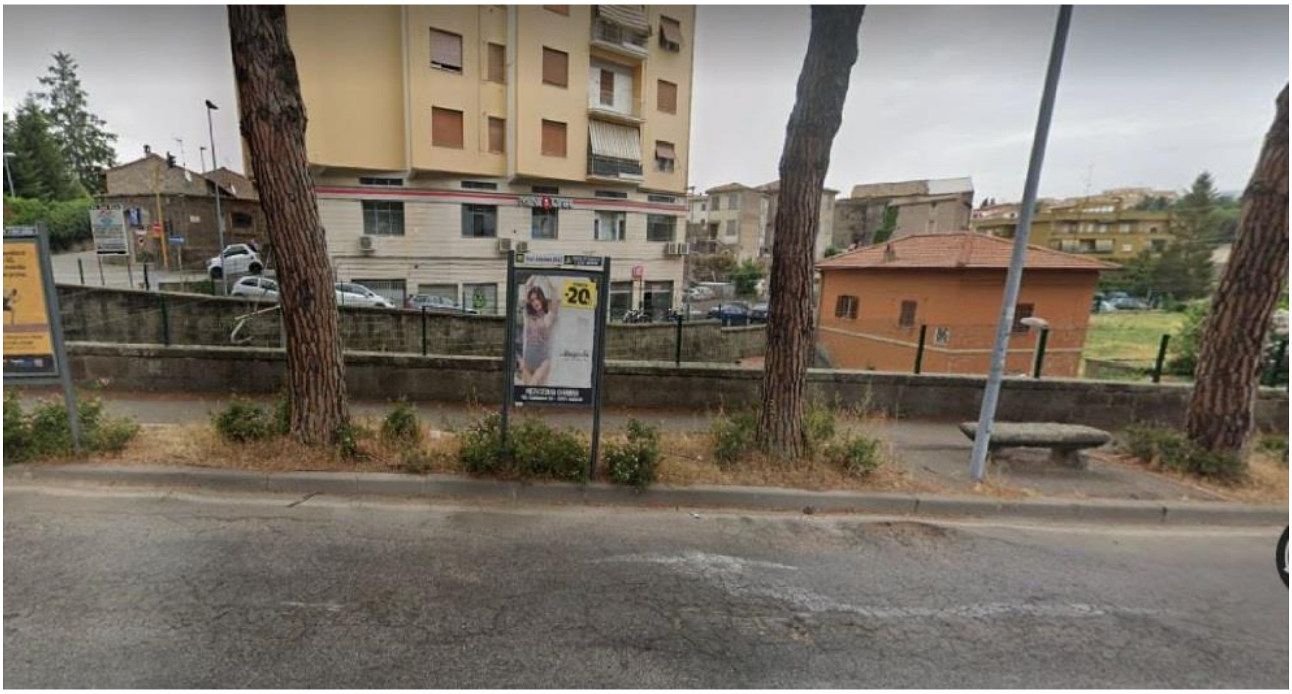

Figure 1. First photo shown in the slideshow of the poor maintenance condition (Source: authors' photographic archive).

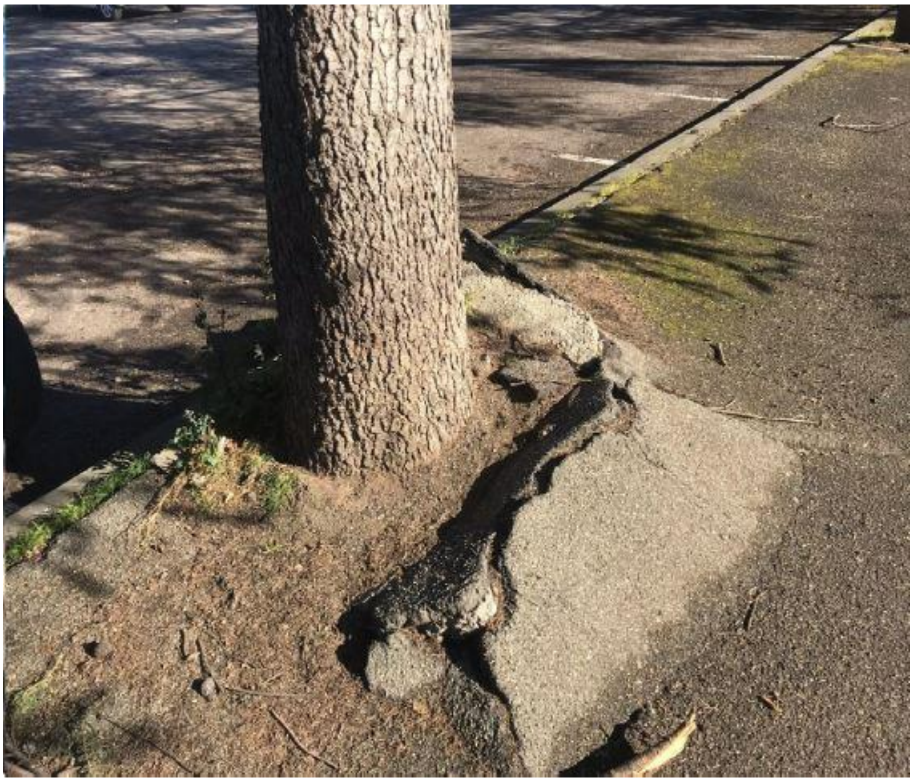

Figure 2. Second photo shown in the slideshow of the poor maintenance condition (Source: authors' photographic archive).

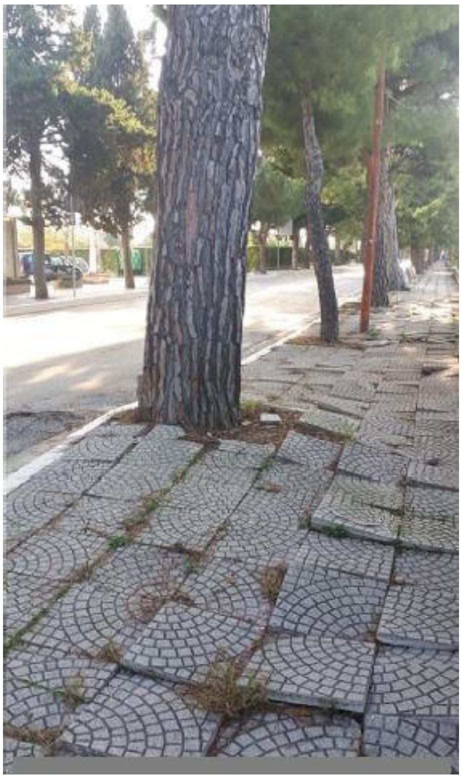

Figure 3. Third photo shown in the slideshow of the poor maintenance condition (Source: Conalpa 2018, http://www.conalpa.it/conoscenza-e-gestione-delle-radici-dei-pini-intervista-allagronomo-naturalista-giovanni-morelli/).

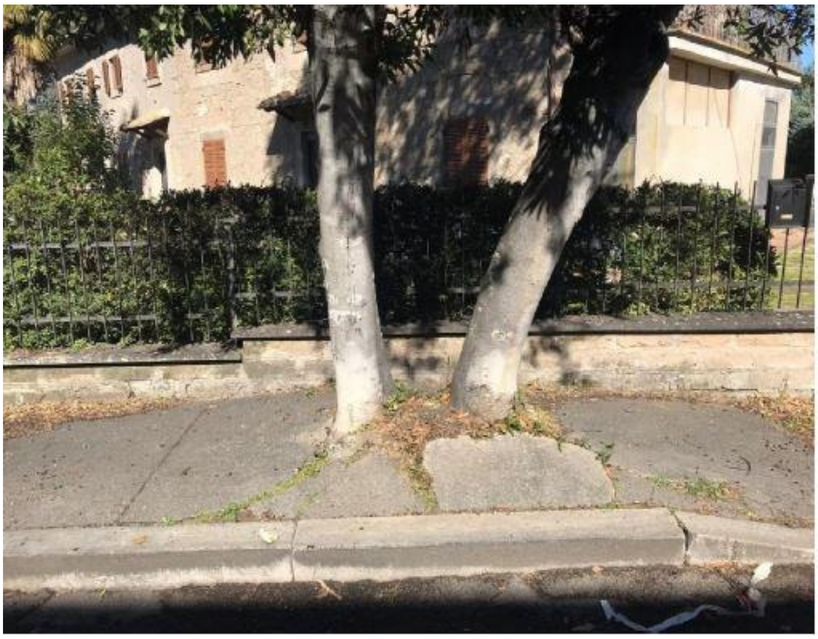

Figure 4. Fourth photo shown in the slideshow of the poor maintenance condition (Source: authors' photographic archive).

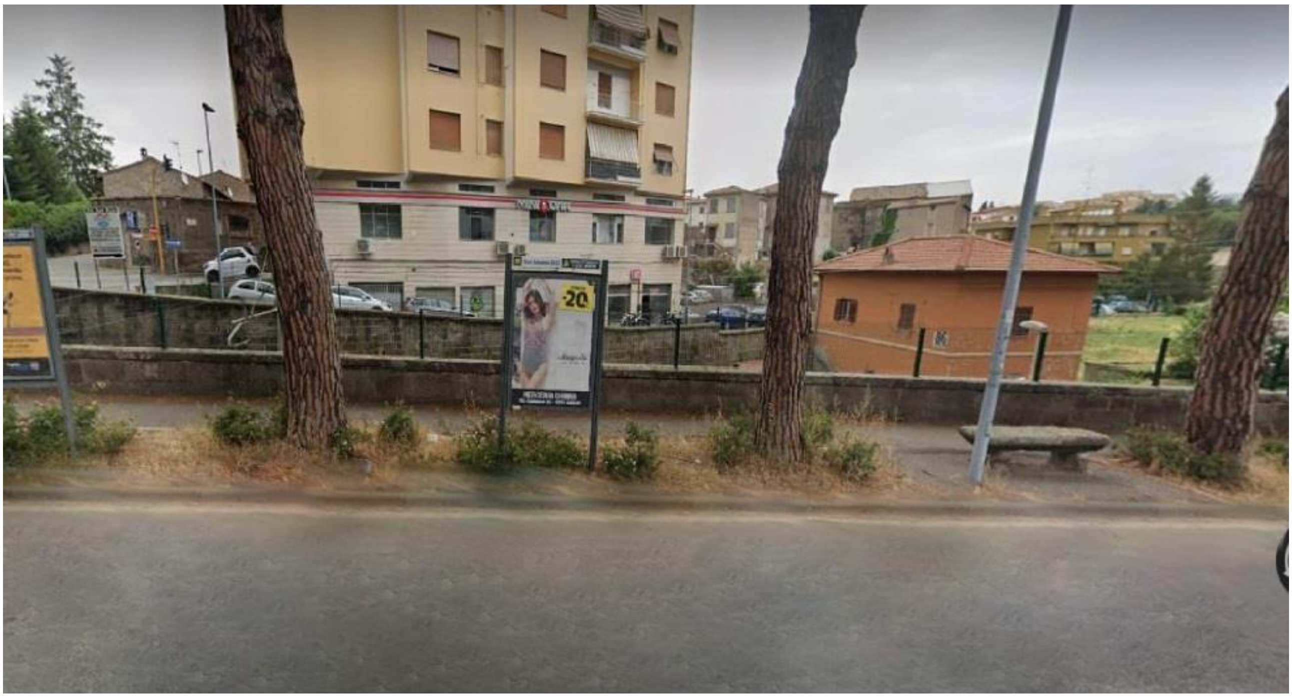

Figure 5. First photo shown in the slideshow of the good maintenance condition (Original source: authors’ photographic archive).

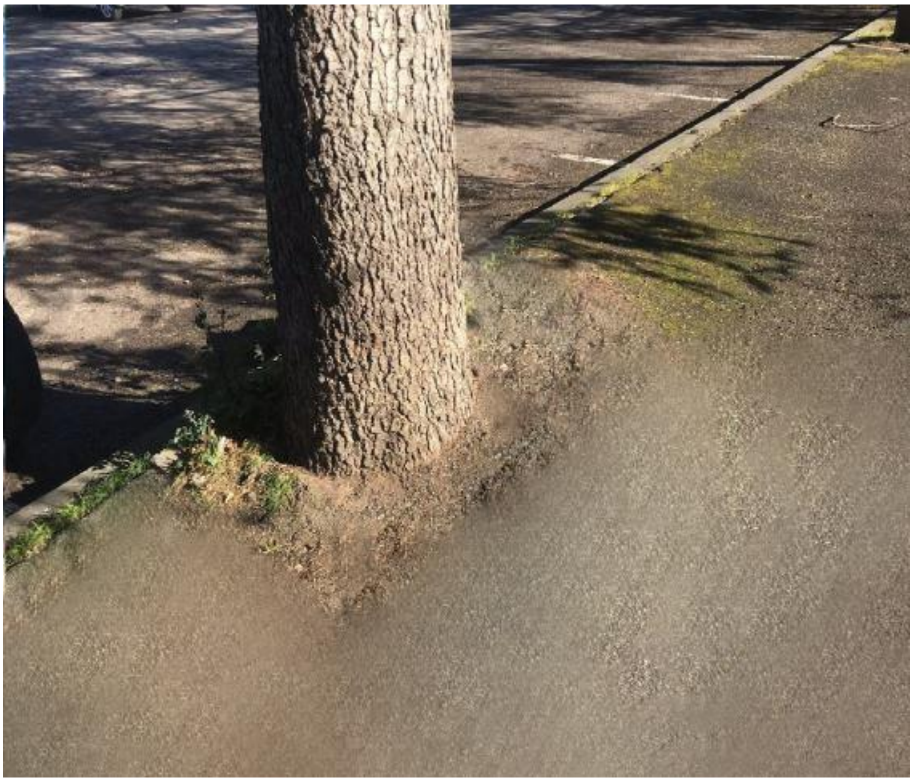

Figure 6. Second photo shown in the slideshow of the good maintenance condition (Original source: authors’ photographic archive).

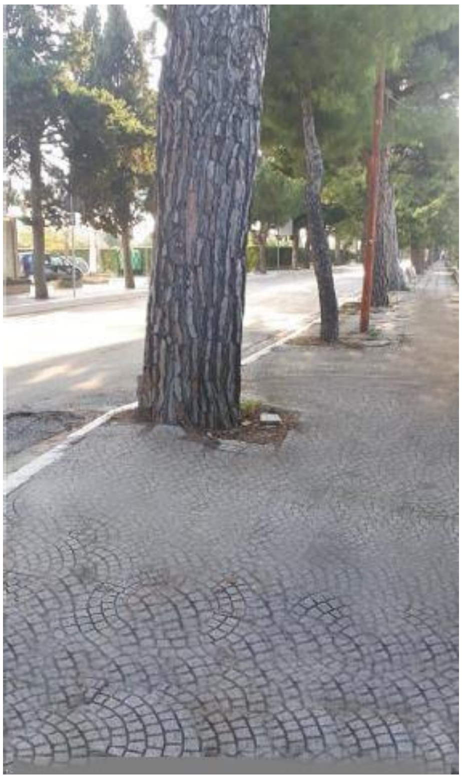

Figure 7. Third photo shown in the slideshow of the good maintenance condition (Original source: Conalpa 2018, http://www.conalpa.it/conoscenza-e-gestione-delle-radici-dei-pini-intervista-allagronomo-naturalista-giovanni-morelli/).

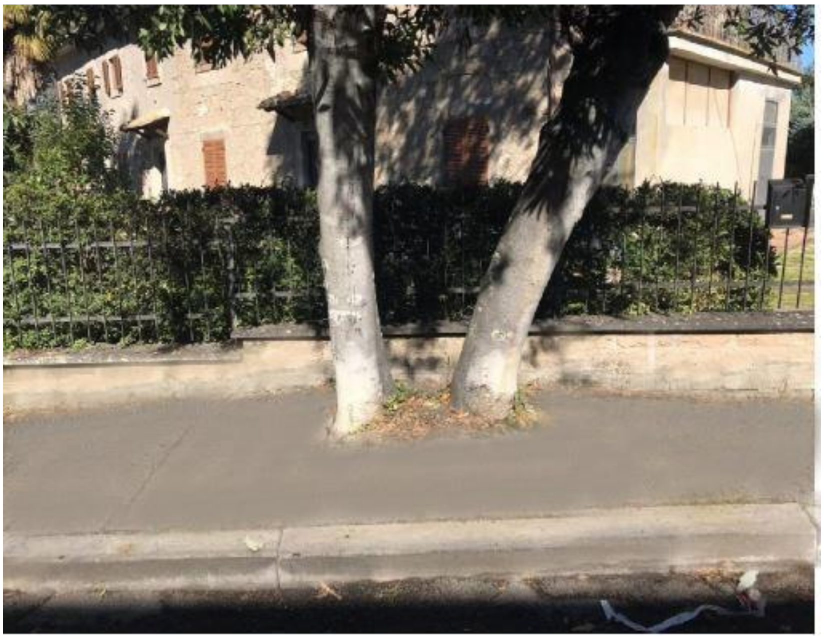

Figure 8. Fourth photo shown in the slideshow of the good maintenance condition (Original source: authors' photographic archive).
